# Supplementary material for: Costa Rican Propolis Chemical Compositions: Nemorosone Found to Be Present in an Exclusive Geographical Zone
Source: Molecules. 2023 Oct 14;28(20):7081. doi: 10.3390/molecules28207081 (PMC10609476; doi:10.3390/molecules28207081)
Supplement: Supplementary file 1 [file molecules-28-07081-s001.zip › molecules-2610481-supplementary.pdf]

Costa Rican Propolis Chemical Compositions: Nemorosone found to be present in an exclusive geographical zone

Eduardo Umaña [1,2,†], Godofredo Solano [1], Gabriel Zamora [2] and Giselle Tamayo-Castillo [1,3,*,†]

1 Centro de Investigaciones en Productos Naturales (CIPRONA), Universidad de Costa Rica, San José 11501 Costa Rica; eduardo.umana.rojas@una.ac.cr (E.U.); godofredo.solano@ucr.ac.cr (G.S.)
2 Centro de Investigaciones Apícolas Tropicales (CINAT), Universidad Nacional, Heredia 3000, Costa Rica; luis.zamora.fallas@una.ac.cr
3 Escuela de Química, Universidad de Costa Rica, San José 11501, Costa Rica
* Correspondence: giselle.tamayo@ucr.ac.cr; Tel.: +506-25112271
† These authors contributed equally to this work.

Table S1: List of samples included in this study and their location information.

Sample	Replicate	Province	Locality	altitude	class	Geographic region	Antioxidant classification	DPPH EC_{50} average
1	A	Alajuela	B. San Jose Norte	948	VCN	Central Valley, North	Low	522.589792
1	B	Alajuela	B. San Jose Norte	948	VCN	Central Valley, North	Low	695.79428
1	C	Alajuela	B. San Jose Norte	948	VCN	Central Valley, North	Low	868.701911
1	D	Alajuela	B. San Jose Norte	948	VCN	Central Valley, North	Low	768.10534
1	E	Alajuela	B. San Jose Norte	948	VCN	Central Valley, North	Low	1099.97556
2	A	Alajuela	Labrador	222	PC	Pacific Coast, Central	Low	304.481999
2	B	Alajuela	Labrador	222	PC	Pacific Coast, Central	Low	910.834132
2	C	Alajuela	Labrador	222	PC	Pacific Coast, Central	Low	642.8918
2	D	Alajuela	Labrador	222	PC	Pacific Coast, Central	Medium	247.601129
2	E	Alajuela	Labrador	222	PC	Pacific Coast, Central	Low	2495.15504
3	A	Alajuela	El Puente	1066	VCN	Central Valley, North	Low	302.972652
3	B	Alajuela	El Puente	1066	VCN	Central Valley, North	Medium	196.73109
3	C	Alajuela	El Puente	1066	VCN	Central Valley, North	Low	1743.53774
3	D	Alajuela	El Puente	1066	VCN	Central Valley, North	High	90.6025291
3	E	Alajuela	El Puente	1066	VCN	Central Valley, North	Low	710.211946

4	A	San Jose	San Martin	122	PC	Pacific Coast, Central	Low	1115.24164
4	B	San Jose	San Martin	122	PC	Pacific Coast, Central	Low	1516.28138
4	C	San Jose	San Martin	122	PC	Pacific Coast, Central	Low	1179.07148
4	D	San Jose	San Martin	122	PC	Pacific Coast, Central	Low	880.626223
4	E	San Jose	San Martin	122	PC	Pacific Coast, Central	Low	1138.24994
5	A	San Jose	San Pedro	282	PC	Pacific Coast, Central	Low	521.256392
5	B	San Jose	San Pedro	282	PC	Pacific Coast, Central	Low	381.800581
5	C	San Jose	San Pedro	282	PC	Pacific Coast, Central	Low	730.346136
5	D	San Jose	San Pedro	282	PC	Pacific Coast, Central	Low	756.528012
5	E	San Jose	San Pedro	282	PC	Pacific Coast, Central	Low	1008.16131
6	A	Guanacaste	Nandayure	70	G	Guanacaste	High	33.8921283
6	B	Guanacaste	Nandayure	70	G	Guanacaste	High	63.1773399
6	C	Guanacaste	Nandayure	70	G	Guanacaste	High	49.8542274
6	D	Guanacaste	Nandayure	70	G	Guanacaste	High	58.4919473
6	E	Guanacaste	Nandayure	70	G	Guanacaste	High	86.0831079
7	A	Guanacaste	Casa	365	G	Guanacaste	Low	453.893932
7	B	Guanacaste	Casa	365	G	Guanacaste	Low	1151.11438

7	C	Guanacaste	Casa	365	G	Guanacaste	Low	753.47304
7	D	Guanacaste	Casa	365	G	Guanacaste	Low	984.759672
7	E	Guanacaste	Casa	365	G	Guanacaste	Low	437.636762
8	B	Guanacaste	Los Cerros	270	G	Guanacaste	Low	1640.97464
8	C	Guanacaste	Los Cerros	270	G	Guanacaste	Low	1719.26519
8	D	Guanacaste	Los Cerros	270	G	Guanacaste	Low	1472.33436
8	E	Guanacaste	Los Cerros	270	G	Guanacaste	Low	1863.20177
9	A	Alajuela	San Jorge	92	ZN	North	High	72.4858618
9	B	Alajuela	San Jorge	92	ZN	North	Low	312.369338
9	C	Alajuela	San Jorge	92	ZN	North	Low	322.557642
9	D	Alajuela	San Jorge	92	ZN	North	Medium	239.405205
9	E	Alajuela	San Jorge	92	ZN	North	High	44.2892768
10	A	Guanacaste	San Jose de la Montana	173	G	Guanacaste	Medium	159.493671
10	B	Guanacaste	San Jose de la Montana	173	G	Guanacaste	High	90.6743185
10	C	Guanacaste	San Jose de la Montana	173	G	Guanacaste	Medium	247.463175
10	D	Guanacaste	San Jose de la Montana	173	G	Guanacaste	Medium	220.066257
10	E	Guanacaste	San Jose de la Montana	173	G	Guanacaste	Low	644.846465
11	A	Guanacaste	Curime	212	G	Guanacaste	Medium	179.517502
11	B	Guanacaste	Curime	212	G	Guanacaste	Medium	144.384271

11	C	Guanacaste	Curime	212	G	Guanacaste	Medium	117.561684
11	D	Guanacaste	Curime	212	G	Guanacaste	Medium	118.490022
11	E	Guanacaste	Curime	212	G	Guanacaste	Low	308.831555
12	A	Guanacaste	El Obispo Mansion	166	G	Guanacaste	Low	2067.43785
12	B	Guanacaste	El Obispo Mansion	166	G	Guanacaste	High	98.3349191
12	C	Guanacaste	El Obispo Mansion	166	G	Guanacaste	High	57.4034077
12	D	Guanacaste	El Obispo Mansion	166	G	Guanacaste	Low	1854.9346
12	E	Guanacaste	El Obispo Mansion	166	G	Guanacaste	Low	2131.69114
13	A	Guanacaste	Coyolito	75	G	Guanacaste	Low	1612.52629
13	B	Guanacaste	Coyolito	75	G	Guanacaste	Low	2171.8823
13	C	Guanacaste	Coyolito	75	G	Guanacaste	Low	1436.09383
13	D	Guanacaste	Coyolito	75	G	Guanacaste	Low	1131.05483
13	E	Guanacaste	Coyolito	75	G	Guanacaste	Low	1571.29456
14	A	San Jose	San Marcos	1514	VCS	Central Valley, South	Low	971.105651
14	B	San Jose	San Marcos	1514	VCS	Central Valley, South	Medium	129.477521
14	C	San Jose	San Marcos	1514	VCS	Central Valley, South	High	57.6110707
14	D	San Jose	San Marcos	1514	VCS	Central Valley, South	High	60.8307909

14	E	San Jose	San Marcos	1514	VCS	Central Valley, South	High	67.1328671
15	A	San Jose	Llano Bonito	1728	VCS	Central Valley, South	Low	347.51704
15	B	San Jose	Llano Bonito	1728	VCS	Central Valley, South	Low	399.317406
15	C	San Jose	Llano Bonito	1728	VCS	Central Valley, South	Low	560.27532
15	D	San Jose	Llano Bonito	1728	VCS	Central Valley, South	Low	774.10349
15	E	San Jose	Llano Bonito	1728	VCS	Central Valley, South	Low	616.831218
16	A	San Jose	Palmichal	939	VCS	Central Valley, South	Low	641.183724
16	B	San Jose	Palmichal	939	VCS	Central Valley, South	Medium	183.698761
16	C	San Jose	Palmichal	939	VCS	Central Valley, South	High	68.4998772
16	D	San Jose	Palmichal	939	VCS	Central Valley, South	Low	427.804029
16	E	San Jose	Palmichal	939	VCS	Central Valley, South	Low	808.823529
17	A	San Jose	San Ignacio	919	VCS	Central Valley, South	Low	738.403135
17	B	San Jose	San Ignacio	919	VCS	Central Valley, South	Medium	113.50201
17	C	San Jose	San Ignacio	919	VCS	Central Valley, South	Low	430.019305
17	D	San Jose	San Ignacio	919	VCS	Central Valley, South	Low	656.474988
17	E	San Jose	San Ignacio	919	VCS	Central Valley, South	Medium	230.06536
18	A	San Jose	Monte Redondo	1075	VCS	Central Valley, South	Low	474.994041

18	B	San Jose	Monte Redondo	1075	VCS	Central Valley, South	Low	867.260901
18	C	San Jose	Monte Redondo	1075	VCS	Central Valley, South	Low	770.898579
18	D	San Jose	Monte Redondo	1075	VCS	Central Valley, South	Medium	289.645185
18	E	San Jose	Monte Redondo	1075	VCS	Central Valley, South	Low	975.847768
19	A	Puntarenas	Santa Elena de Pittier	1030	ZS	South	High	47.7944627
19	B	Puntarenas	Santa Elena de Pittier	1030	ZS	South	High	71.8647252
19	C	Puntarenas	Santa Elena de Pittier	1030	ZS	South	High	41.2865074
19	D	Puntarenas	Santa Elena de Pittier	1030	ZS	South	High	67.8362573
19	E	Puntarenas	Santa Elena de Pittier	1030	ZS	South	High	53.5149599
20	A	Puntarenas	Cebrol	357	ZS	South	High	67.6513317
20	B	Puntarenas	Cebrol	357	ZS	South	High	81.4254332
20	C	Puntarenas	Cebrol	357	ZS	South	High	81.3679245
20	D	Puntarenas	Cebrol	357	ZS	South	High	79.5606495
20	E	Puntarenas	Cebrol	357	ZS	South	Medium	224.340176
21*	A	Guanacaste	San Pedro-Luis	71	G	Guanacaste	Medium	218.004338
21*	B	Guanacaste	San Pedro-Luis	71	G	Guanacaste	Low	2338.08744
21*	C	Guanacaste	San Pedro-Luis	71	G	Guanacaste	Low	388.845585
21*	D	Guanacaste	San Pedro-Luis	71	G	Guanacaste	High	83.6163597
21*	E	Guanacaste	San Pedro-Luis	71	G	Guanacaste	Low	1421.64898
22*	A	San Jose	Pejivalle	735	ZS	South	High	99.0318772
22*	B	San Jose	Pejivalle	735	ZS	South	High	83.4714773
22*	C	San Jose	Pejivalle	735	ZS	South	High	91.1692156
22*	D	San Jose	Pejivalle	735	ZS	South	High	69.4410244

22*	E	San Jose	Pejivalle	735	ZS	South	High	92.3407301
23*	A	San Jose	La Potenciana	1340	PC	Pacific Coast, Central	High	87.1756857
23*	B	San Jose	La Potenciana	1340	PC	Pacific Coast, Central	Medium	105.211647
23*	C	San Jose	La Potenciana	1340	PC	Pacific Coast, Central	High	46.9273743
23*	D	San Jose	La Potenciana	1340	PC	Pacific Coast, Central	High	96.8757568
23*	E	San Jose	La Potenciana	1340	PC	Pacific Coast, Central	High	84.0137728
24*	A	San Jose	Tarbaca	1526	VCS	Central Valley, South	High	84.9615754
24*	B	San Jose	Tarbaca	1526	VCS	Central Valley, South	Low	376.972606
24*	C	San Jose	Tarbaca	1526	VCS	Central Valley, South	Medium	111.492862
24*	D	San Jose	Tarbaca	1526	VCS	Central Valley, South	Medium	115.89481
24*	E	San Jose	Tarbaca	1526	VCS	Central Valley, South	Medium	207.26257

*Samples use for validation of PLS-DA model.

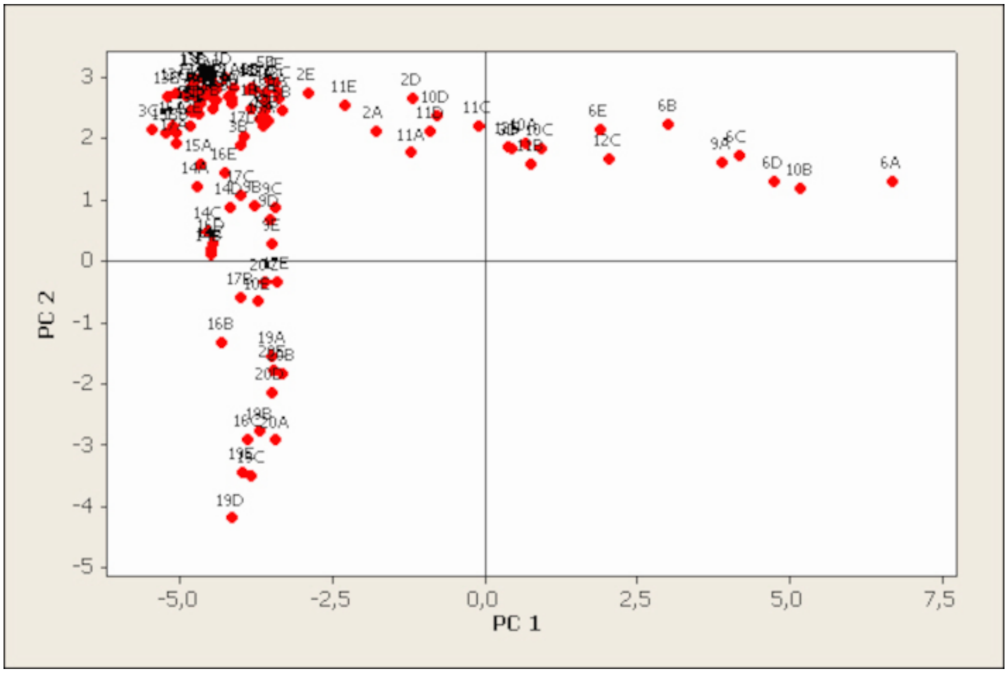

Figure S1. PCA obtained for 99 propolis samples using NMR bins from nemorosone and *n*-coniferyl benzoate.

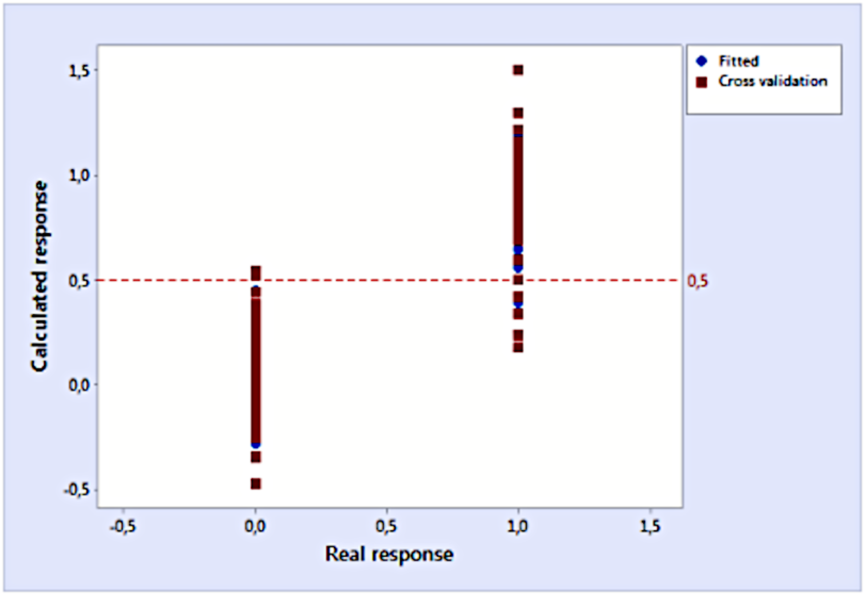

Figure S2: Discriminant model predictive evaluation of 99 propolis samples by cross validation, using NMR bins from nemorosone and *n*-coniferyl benzoate.

Next figures: NMR spectra and HRMS spectra of isolated compounds

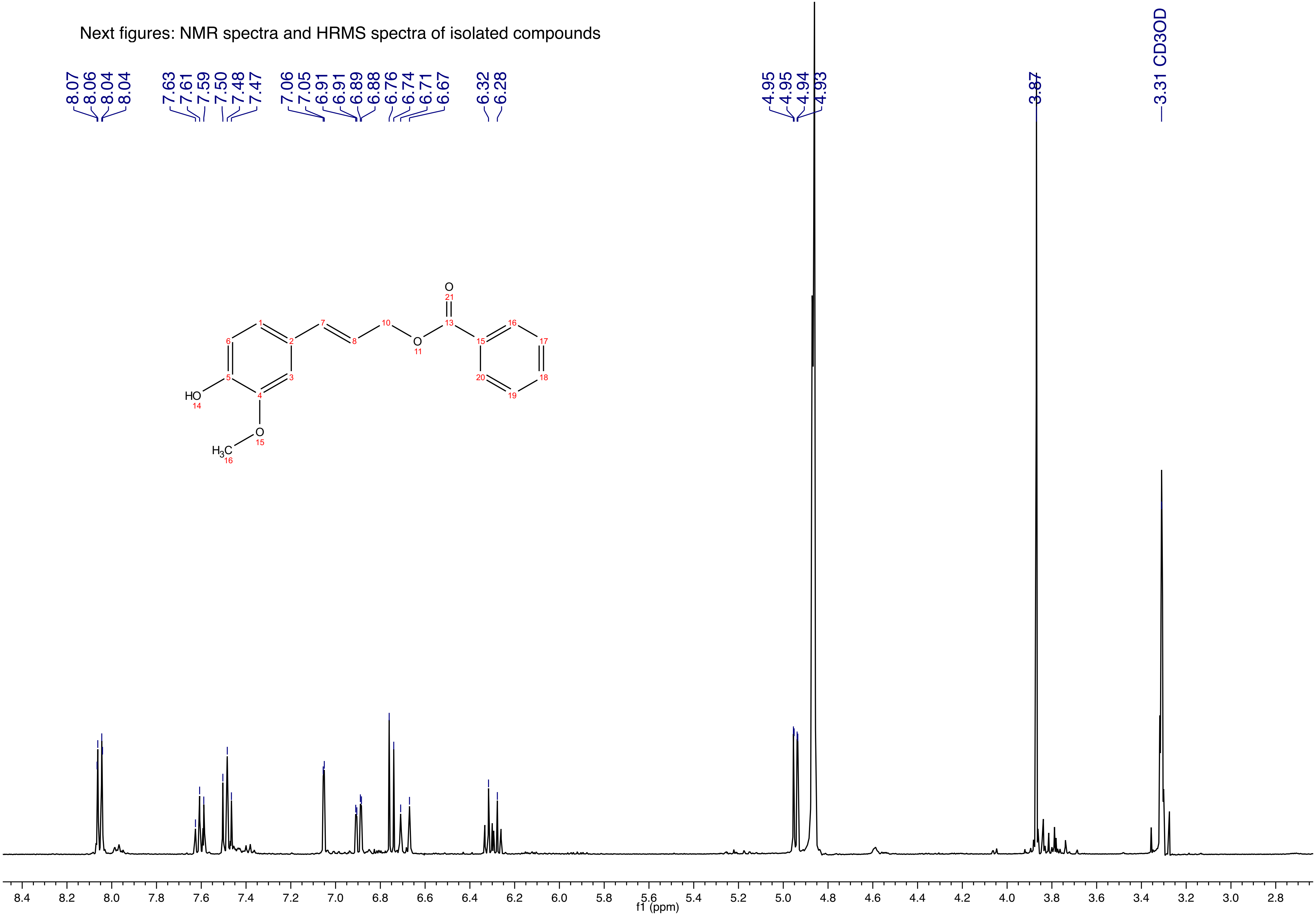

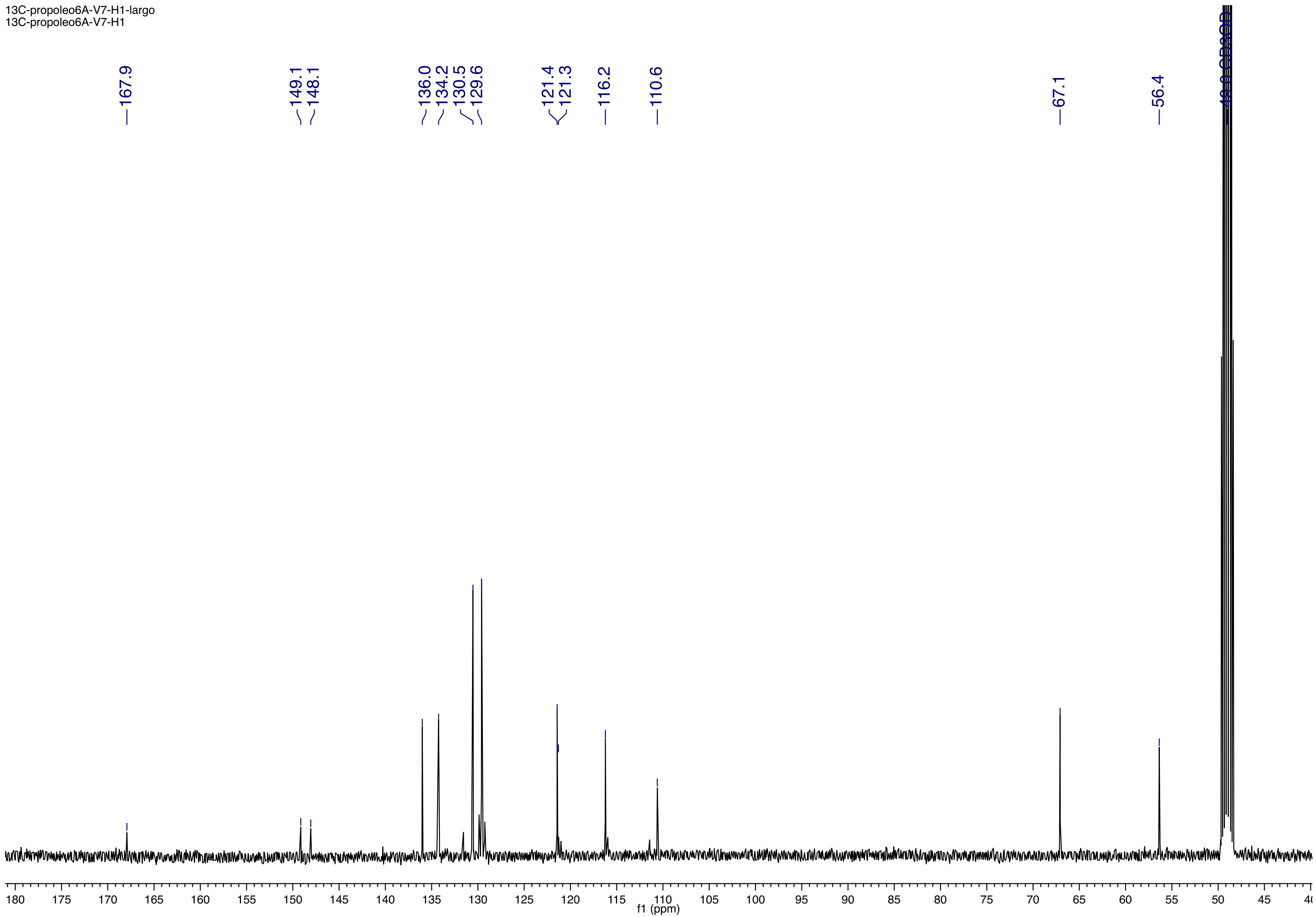
13C-propoleo6A-V7-H1-largo
13C-propoleo6A-V7-H1
167.9
149.1
148.1
136.0
134.2
130.5
129.6
121.4
121.3
116.2
110.6
67.1
56.4
49.0 CD3OD
180
175
170
165
160
155
150
145
140
135
130
125
120
115
110
105
100
95
90
85
80
75
70
65
60
55
50
45
f1 (ppm)

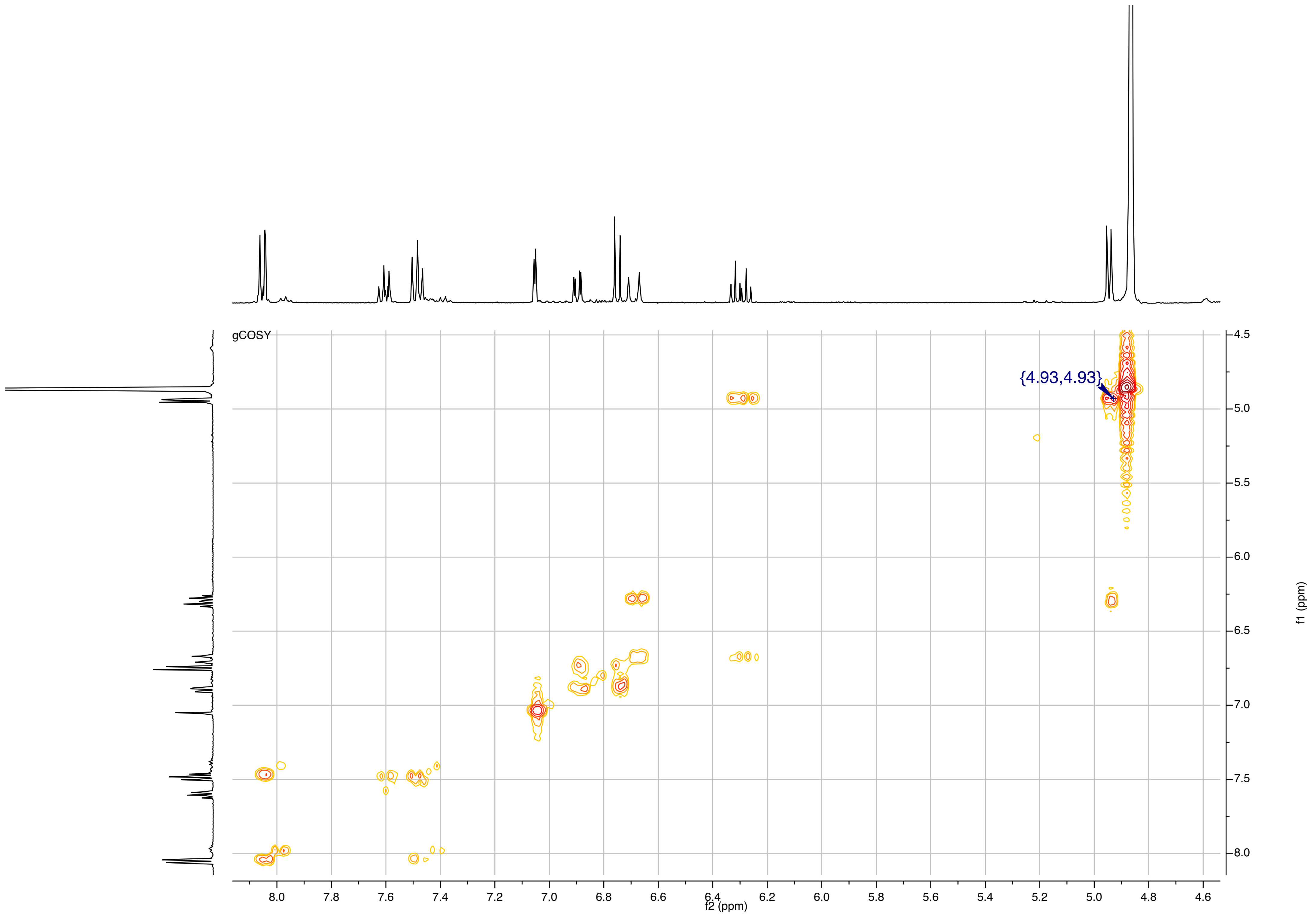
gCOSY
{4.93,4.93}
f2 (ppm)
f1 (ppm)
8.0
7.8
7.6
7.4
7.2
7.0
6.8
6.6
6.4
6.2
6.0
5.8
5.6
5.4
5.2
5.0
4.8
4.6
4.5
5.0
5.5
6.0
6.5
7.0
7.5
8.0

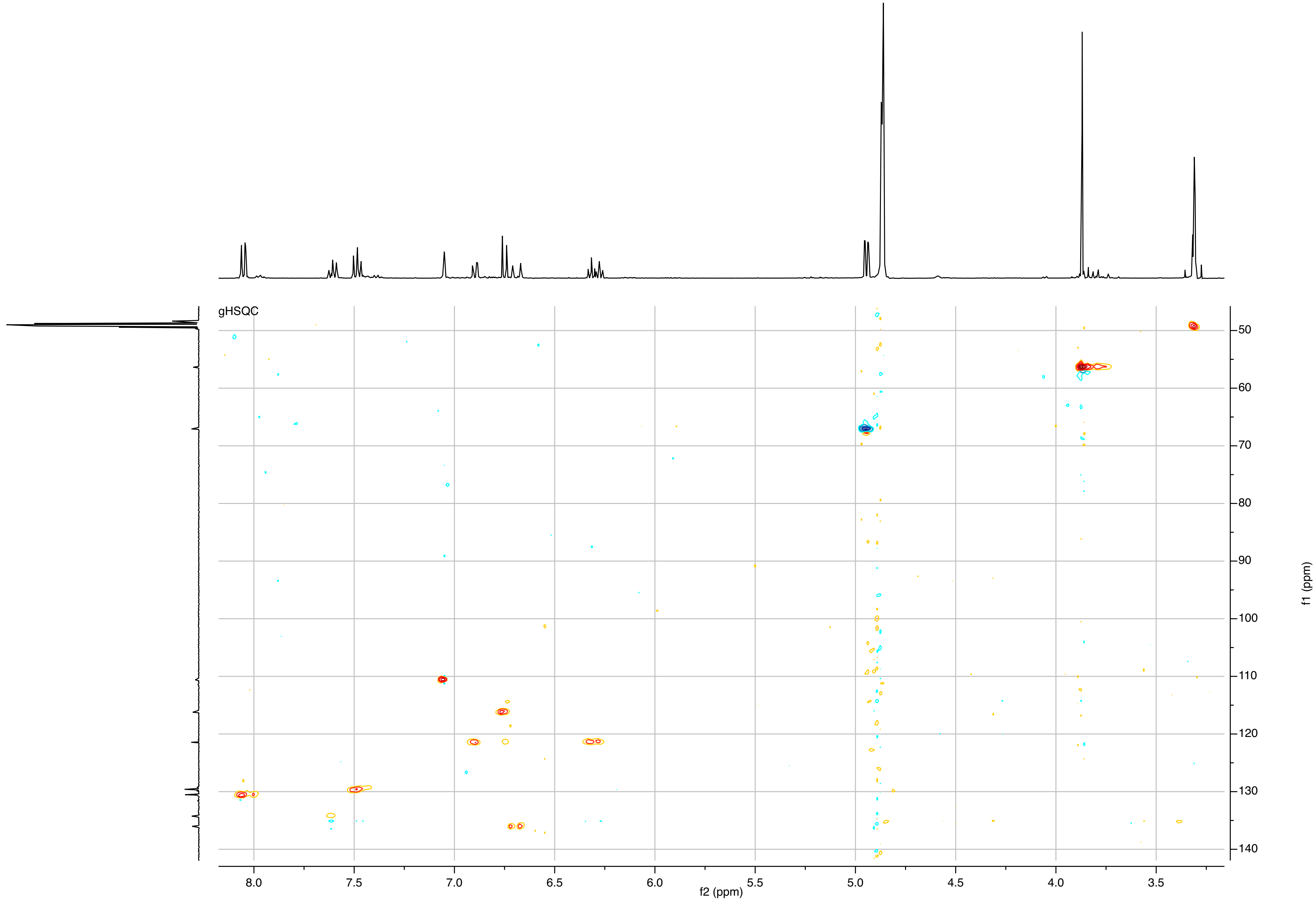
gHSQC
f2 (ppm)
f1 (ppm)
8.0
7.5
7.0
6.5
6.0
5.5
5.0
4.5
4.0
3.5
50
60
70
80
90
100
110
120
130
140

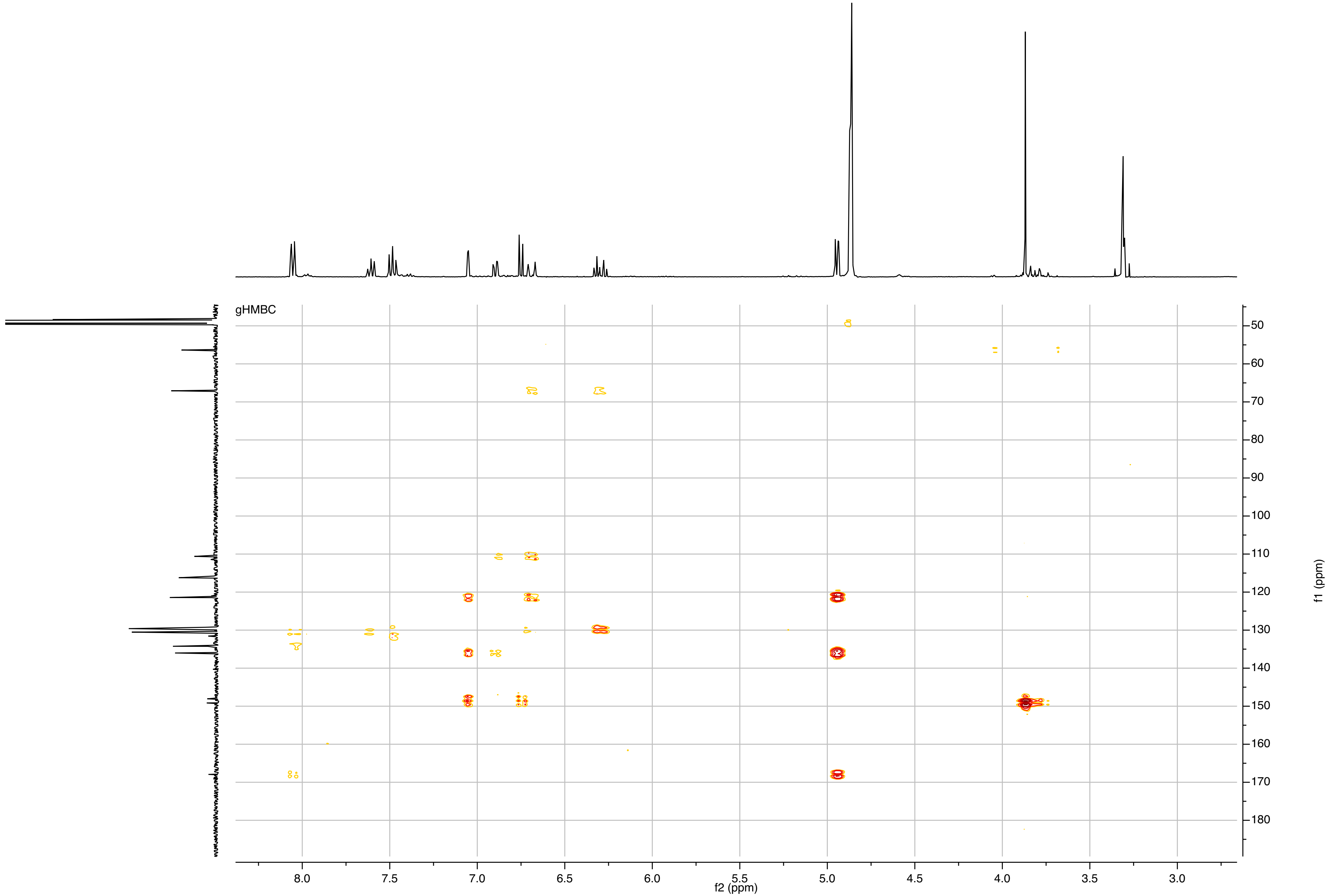

gHMBC
f2 (ppm)
f1 (ppm)
8.0
7.5
7.0
6.5
6.0
5.5
5.0
4.5
4.0
3.5
3.0
50
60
70
80
90
100
110
120
130
140
150
160
170
180

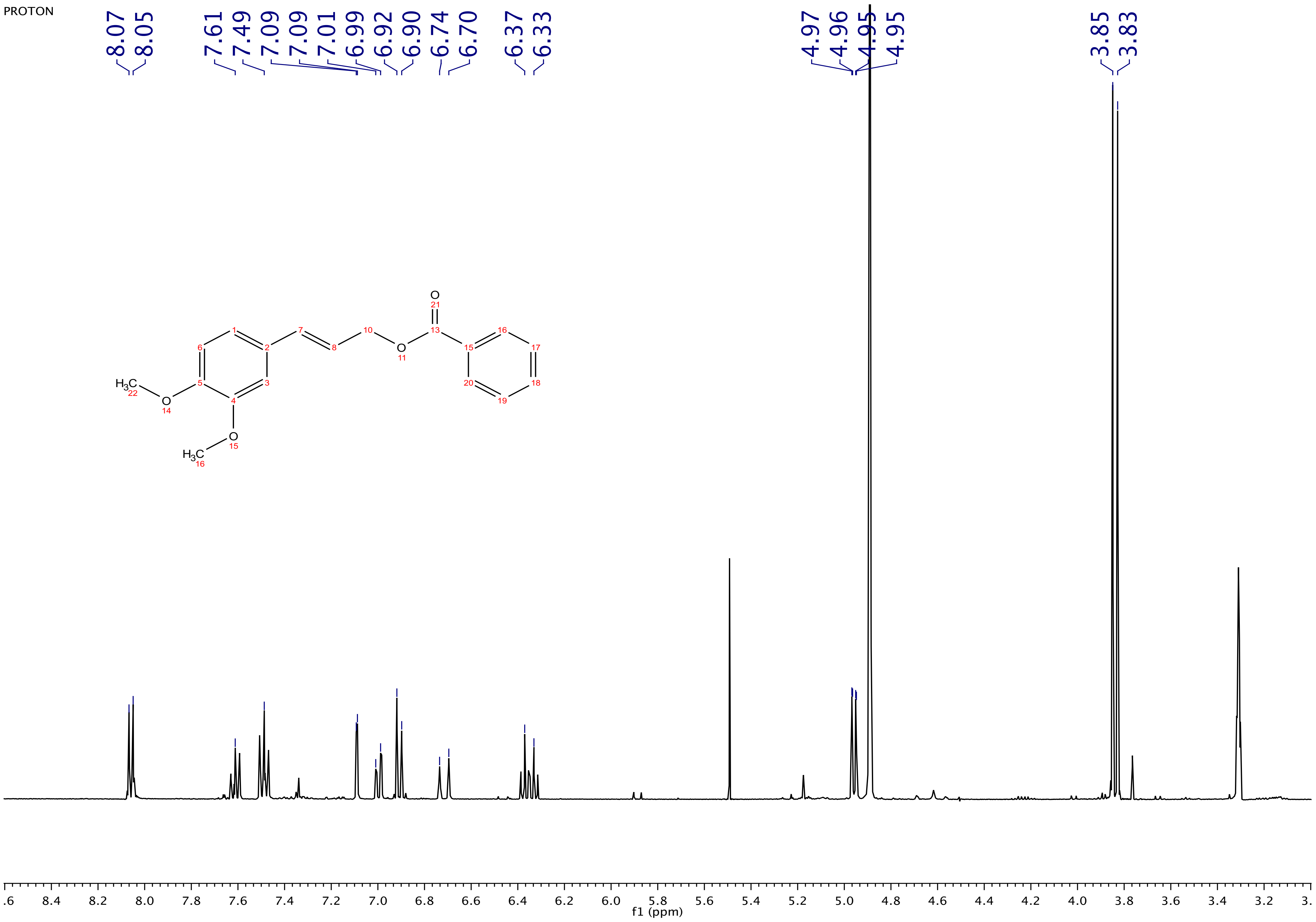
PROTON
8.07
8.05
7.61
7.49
7.09
7.09
7.01
6.99
6.92
6.90
6.74
6.70
6.37
6.33
4.97
4.96
4.95
4.95
3.85
3.83
O
H3C
O
O
H3C
O
.6 8.4 8.2 8.0 7.8 7.6 7.4 7.2 7.0 6.8 6.6 6.4 6.2 6.0 5.8 5.6 5.4 5.2 5.0 4.8 4.6 4.4 4.2 4.0 3.8 3.6 3.4 3.2 3.
f1 (ppm)

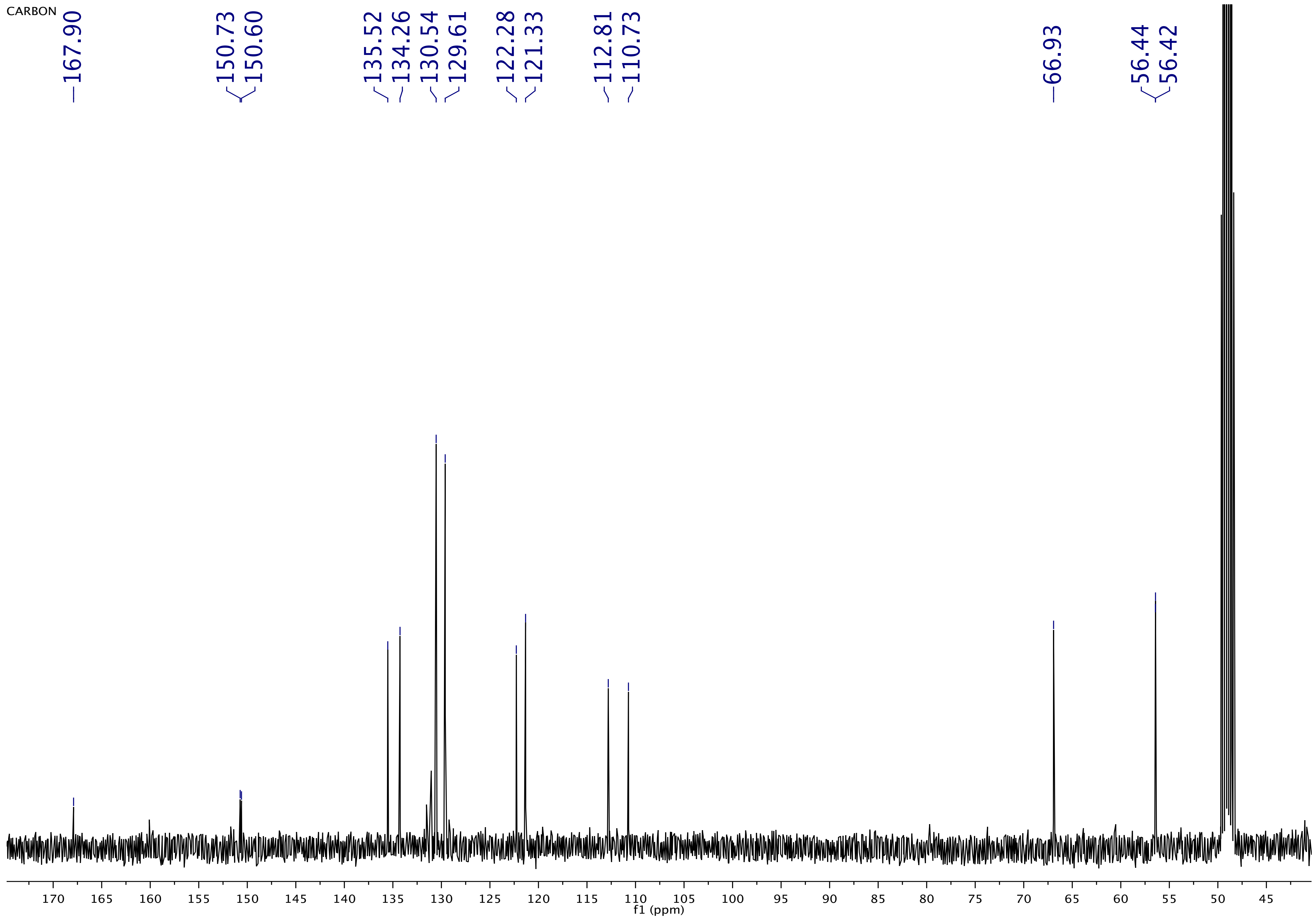
CARBON
167.90
150.73
150.60
135.52
134.26
130.54
129.61
122.28
121.33
112.81
110.73
66.93
56.44
56.42
170
165
160
155
150
145
140
135
130
125
120
115
110
105
100
95
90
85
80
75
70
65
60
55
50
45
f1 (ppm)

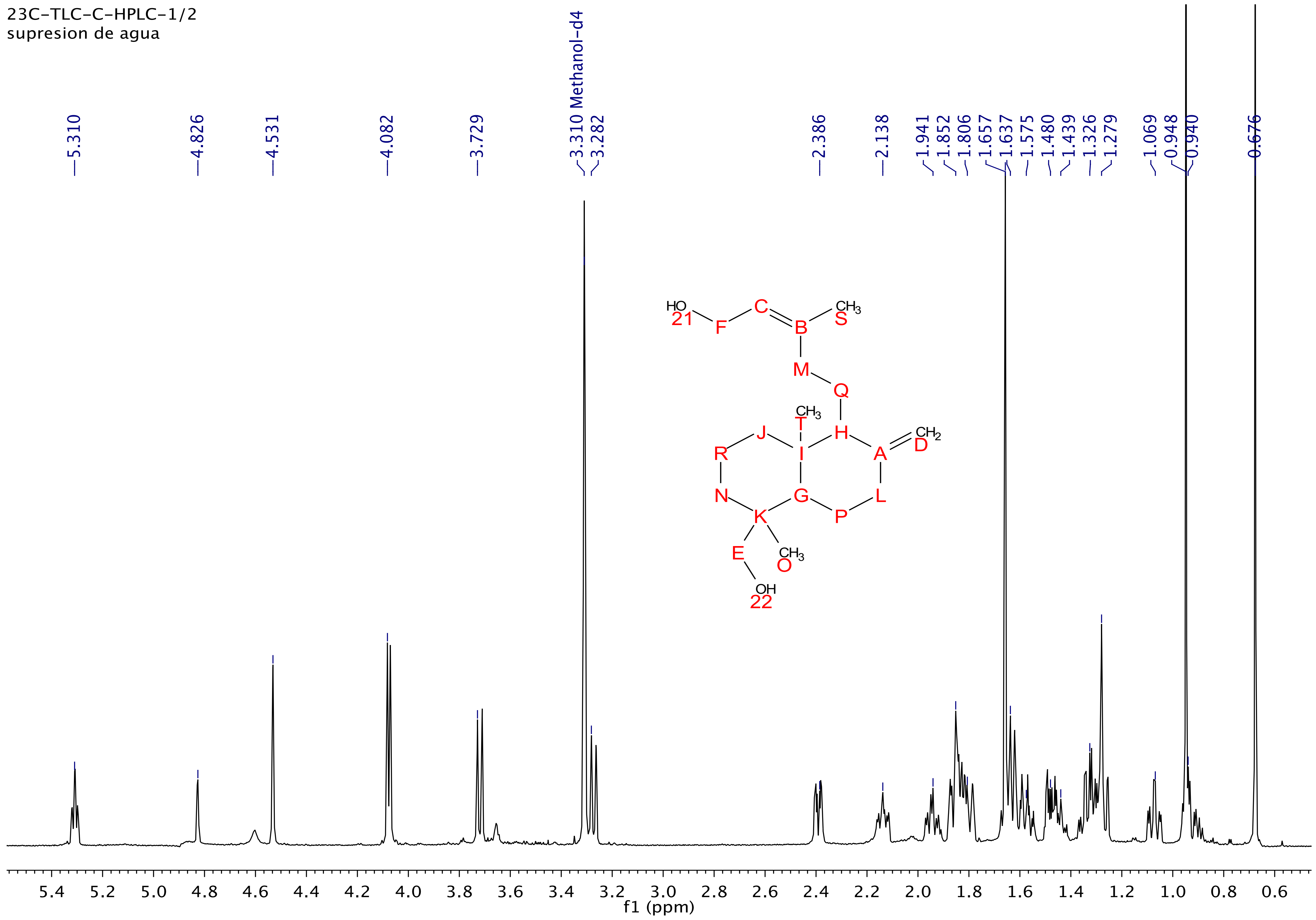
23C-TLC-C-HPLC-1/2
supresion de agua
5.310
4.826
4.531
4.082
3.729
3.310 Methanol-d4
3.282
2.386
2.138
1.941
1.852
1.806
1.657
1.637
1.575
1.480
1.439
1.326
1.279
1.069
0.948
0.940
0.676
HO
21
F
C
B
CH3
S
M
Q
CH3
T
J
I
H
A
CH2
D
R
N
G
L
K
P
E
CH3
O
OH
22
5.4 5.2 5.0 4.8 4.6 4.4 4.2 4.0 3.8 3.6 3.4 3.2 3.0 2.8 2.6 2.4 2.2 2.0 1.8 1.6 1.4 1.2 1.0 0.8 0.6
f1 (ppm)

23C–TLC–C–HPLC–1/5
13C

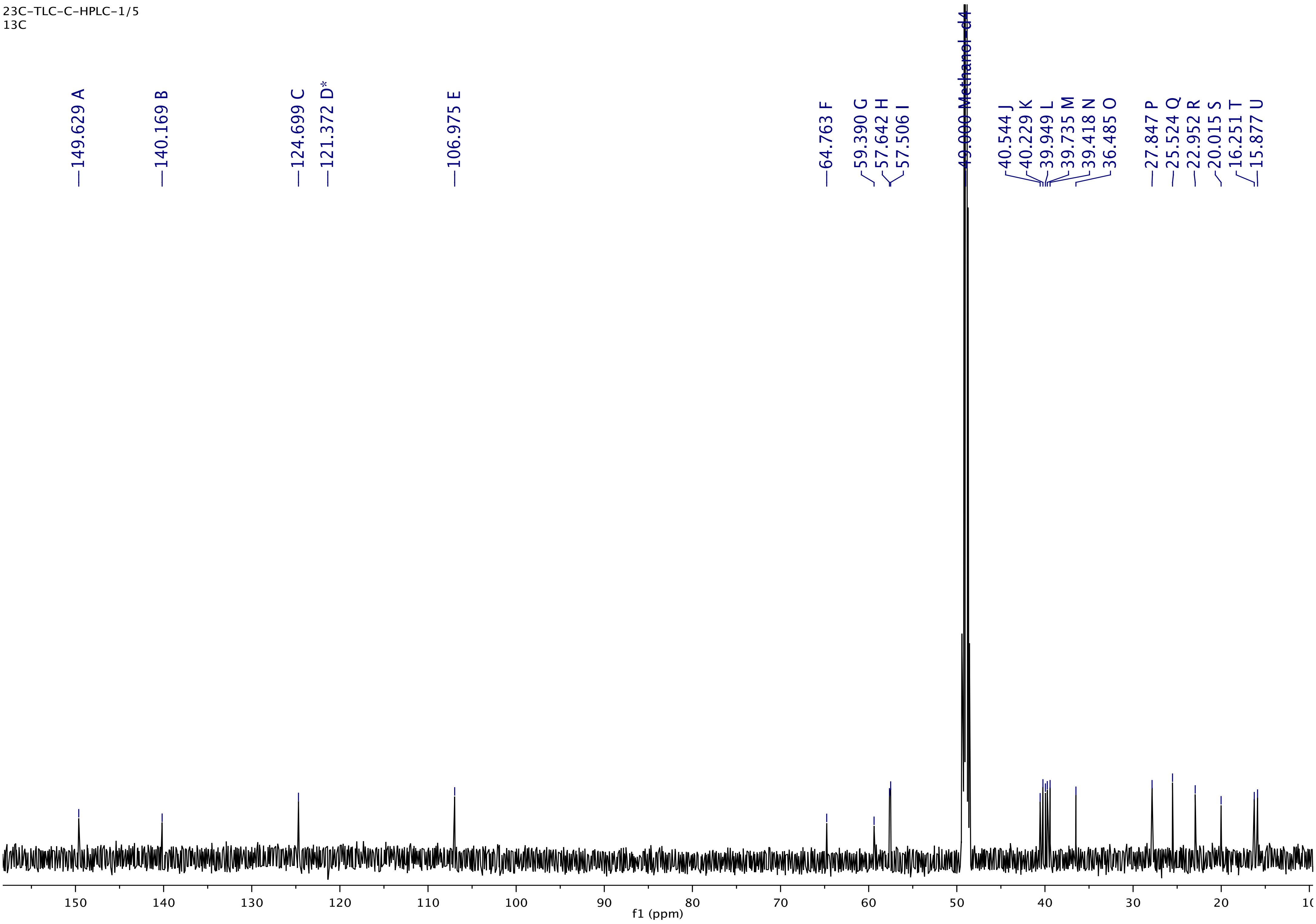

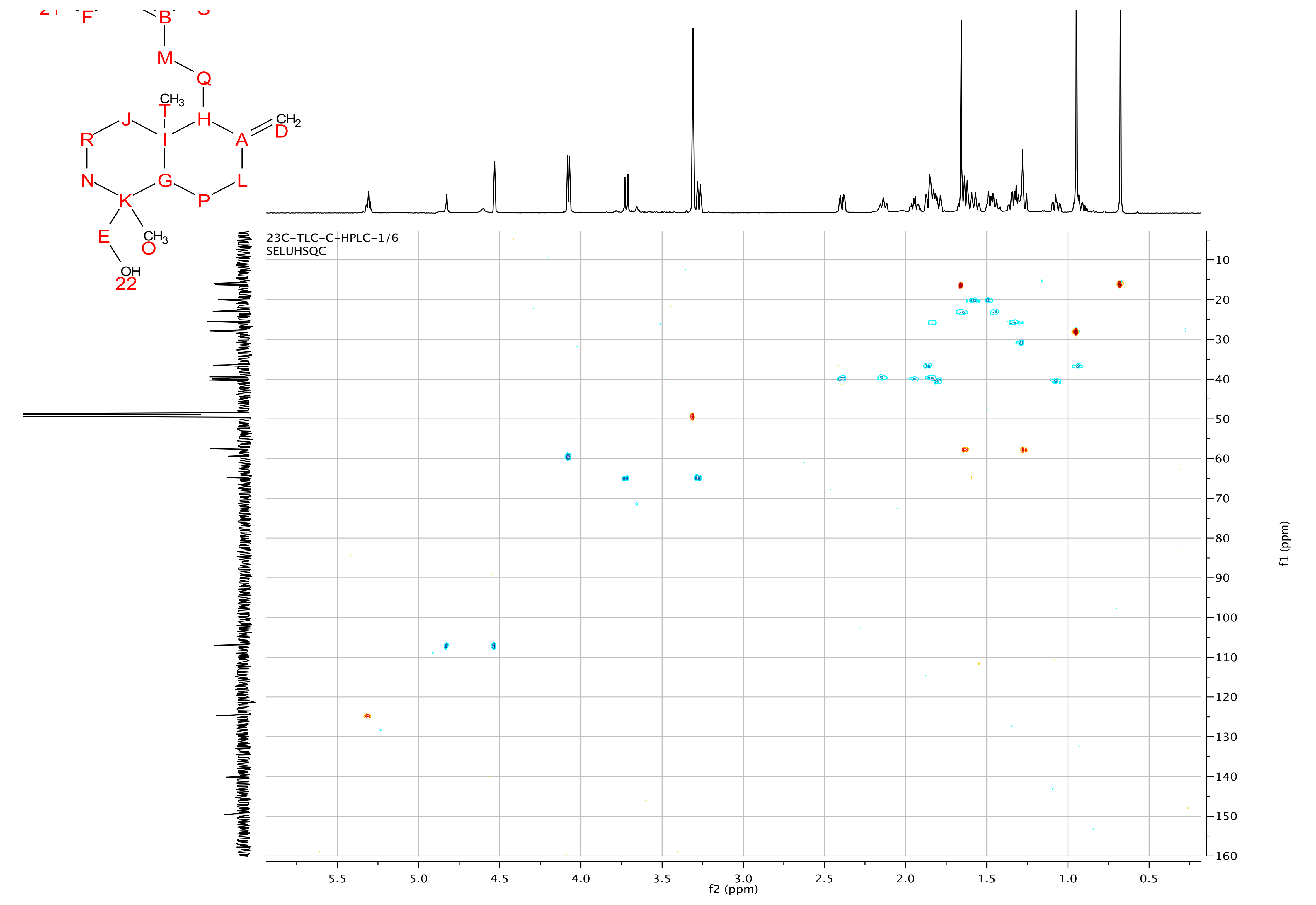

23C-TLC-C-HPLC-1/6
SELUHSQC
f2 (ppm)
f1 (ppm)
CH3
CH2
OH
22

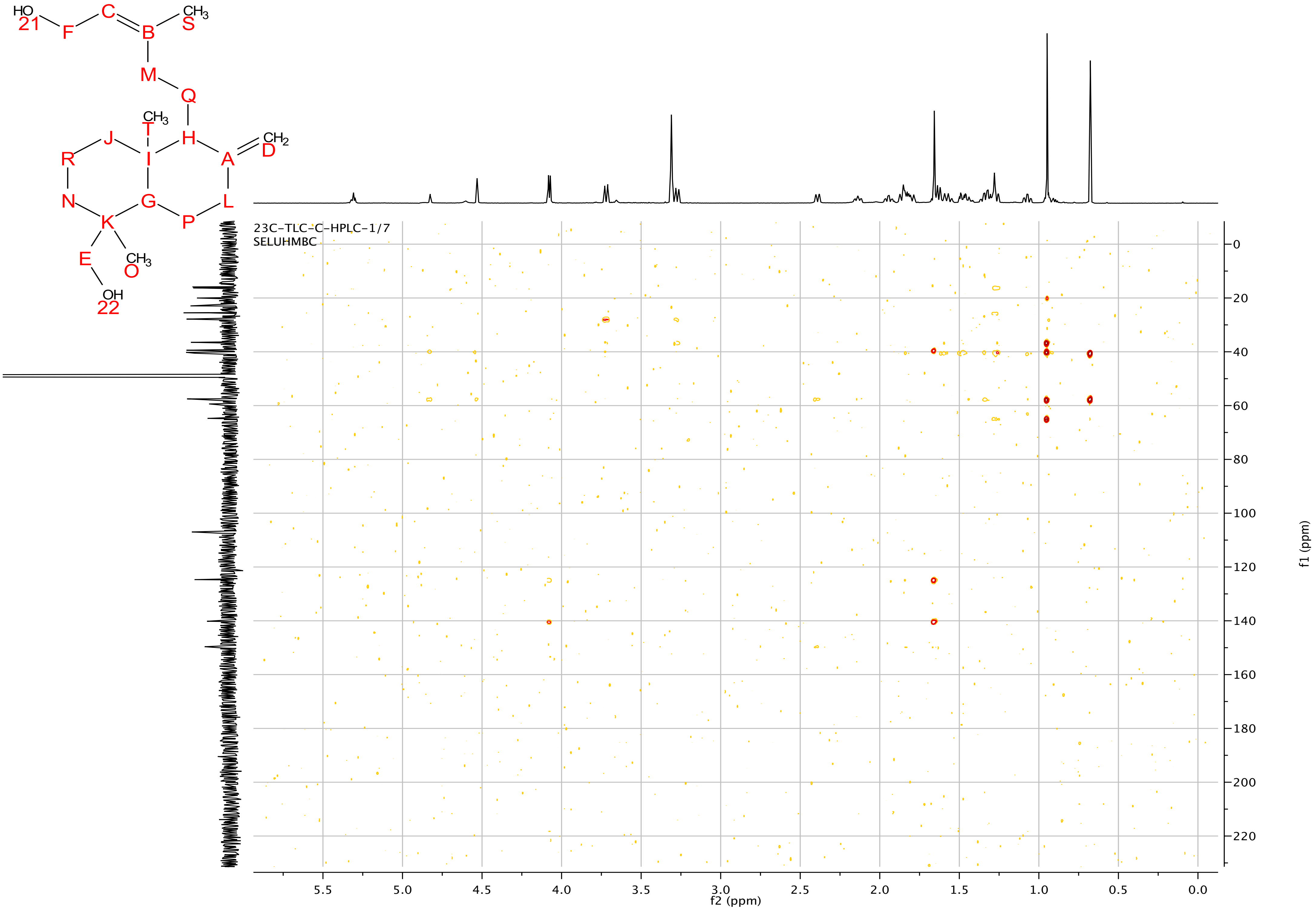
HO
21
F
C
B
CH3
S
M
Q
CH3
T
J
H
CH2
D
R
I
A
N
G
L
K
P
E
CH3
O
OH
22
23C-TLC-C-HPLC-1/7
SELUHMBC
f2 (ppm)
f1 (ppm)

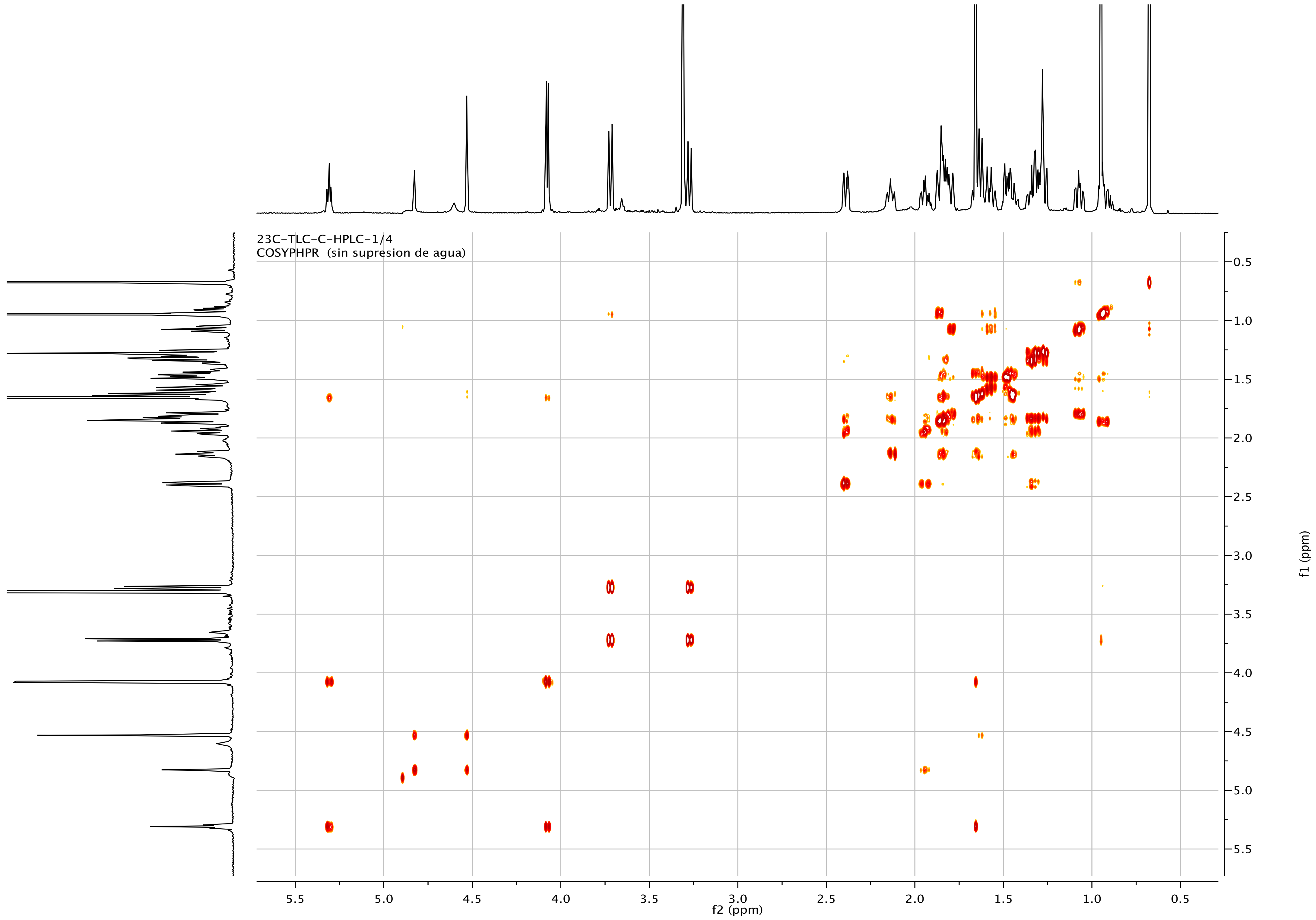

23C-TLC-C-HPLC-1/4
COSYPHPR (sin supresion de agua)
f2 (ppm)
f1 (ppm)
5.5
5.0
4.5
4.0
3.5
3.0
2.5
2.0
1.5
1.0
0.5

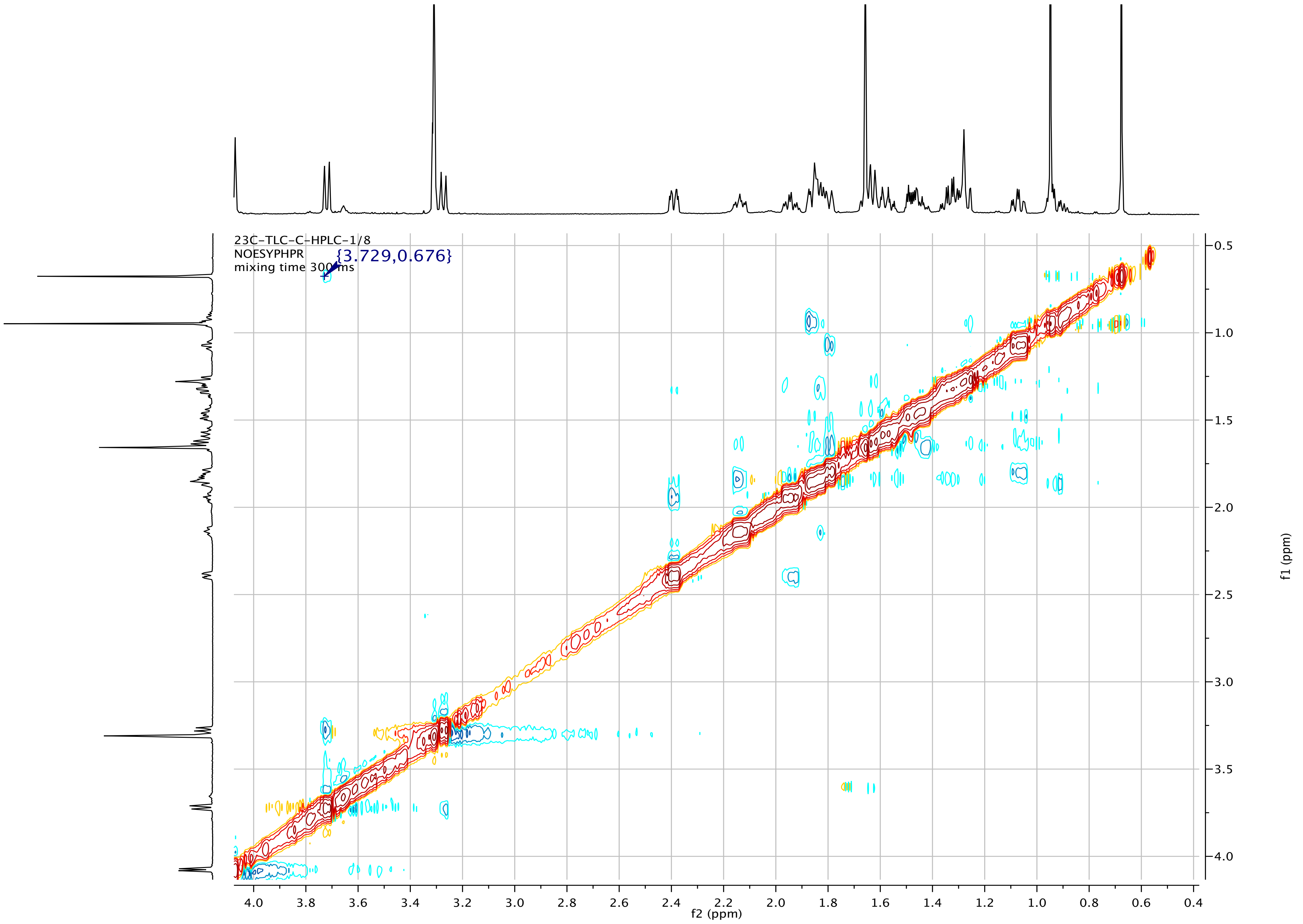
23C-TLC-C-HPLC-1/8
NOESYPHPR
mixing time 300 ms
{3.729,0.676}
f2 (ppm)
f1 (ppm)

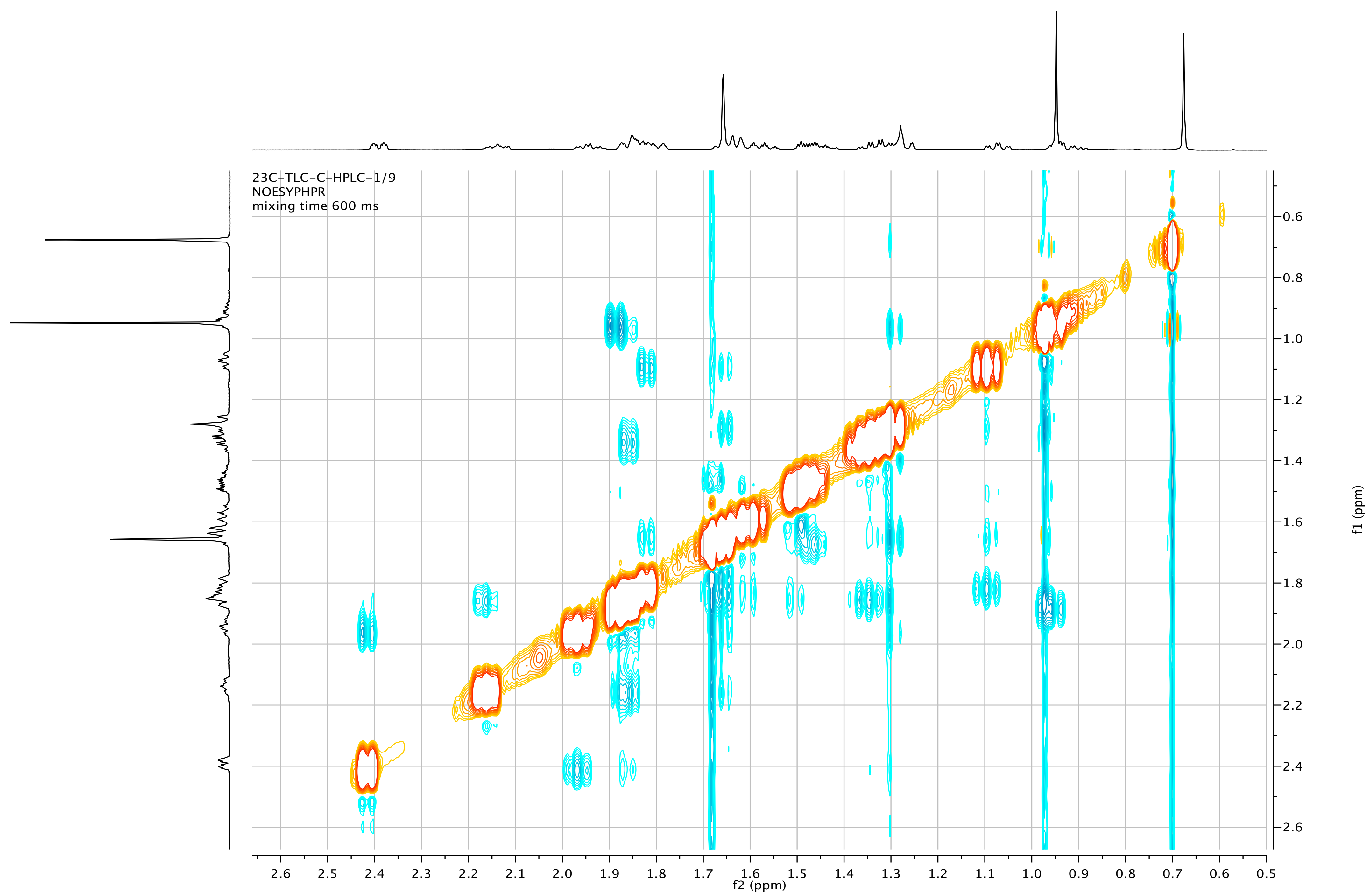

23C-TLC-C-HPLC-1/9
NOESYPHPR
mixing time 600 ms
f2 (ppm)
f1 (ppm)

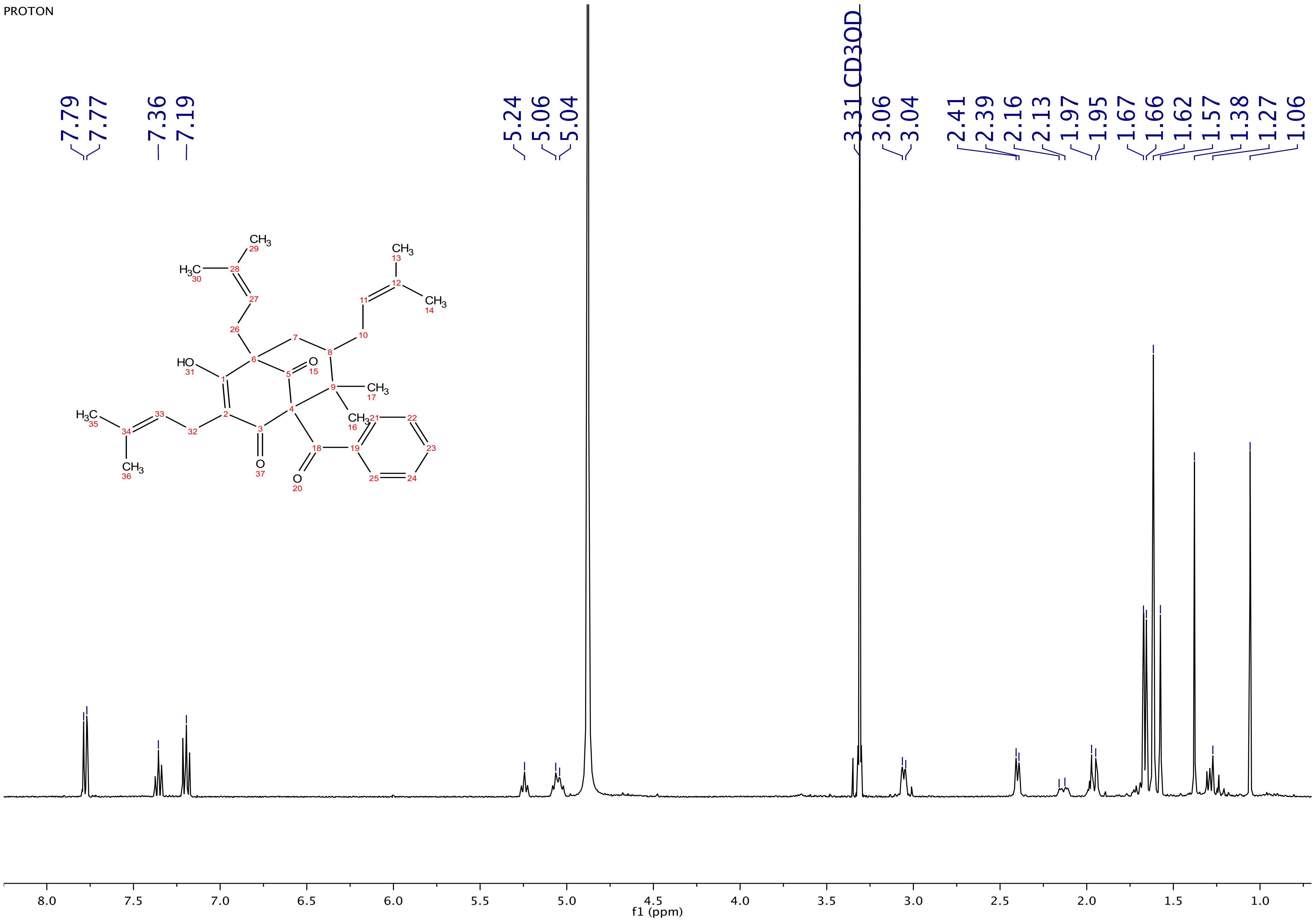
PROTON
7.79
7.77
7.36
7.19
5.24
5.06
5.04
3.31 CD3OD
3.06
3.04
2.41
2.39
2.16
2.13
1.97
1.95
1.67
1.66
1.62
1.57
1.38
1.27
1.06
CH3
H3C
CH3
CH3
HO
O
CH3
H3C
CH3
O
O
CH3
8.0
7.5
7.0
6.5
6.0
5.5
5.0
4.5
4.0
3.5
3.0
2.5
2.0
1.5
1.0
f1 (ppm)

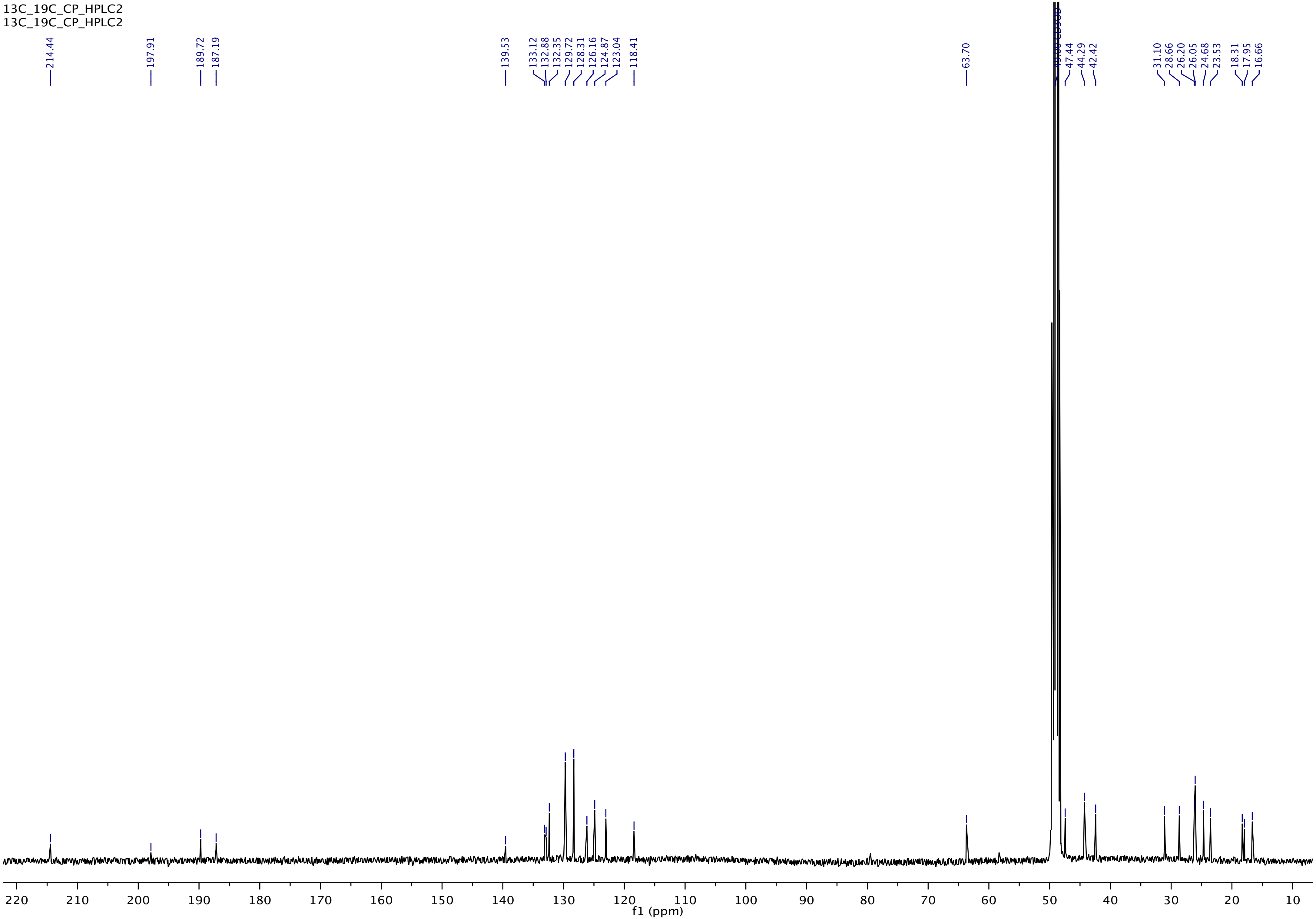
13C_19C_CP_HPLC2
13C_19C_CP_HPLC2
214.44
197.91
189.72
187.19
139.53
133.12
132.88
132.35
129.72
128.31
126.16
124.87
123.04
118.41
63.70
47.44
44.29
42.42
31.10
28.66
26.20
26.05
24.68
23.53
18.31
17.95
16.66
220 210 200 190 180 170 160 150 140 130 120 110 100 90 80 70 60 50 40 30 20 10
f1 (ppm)

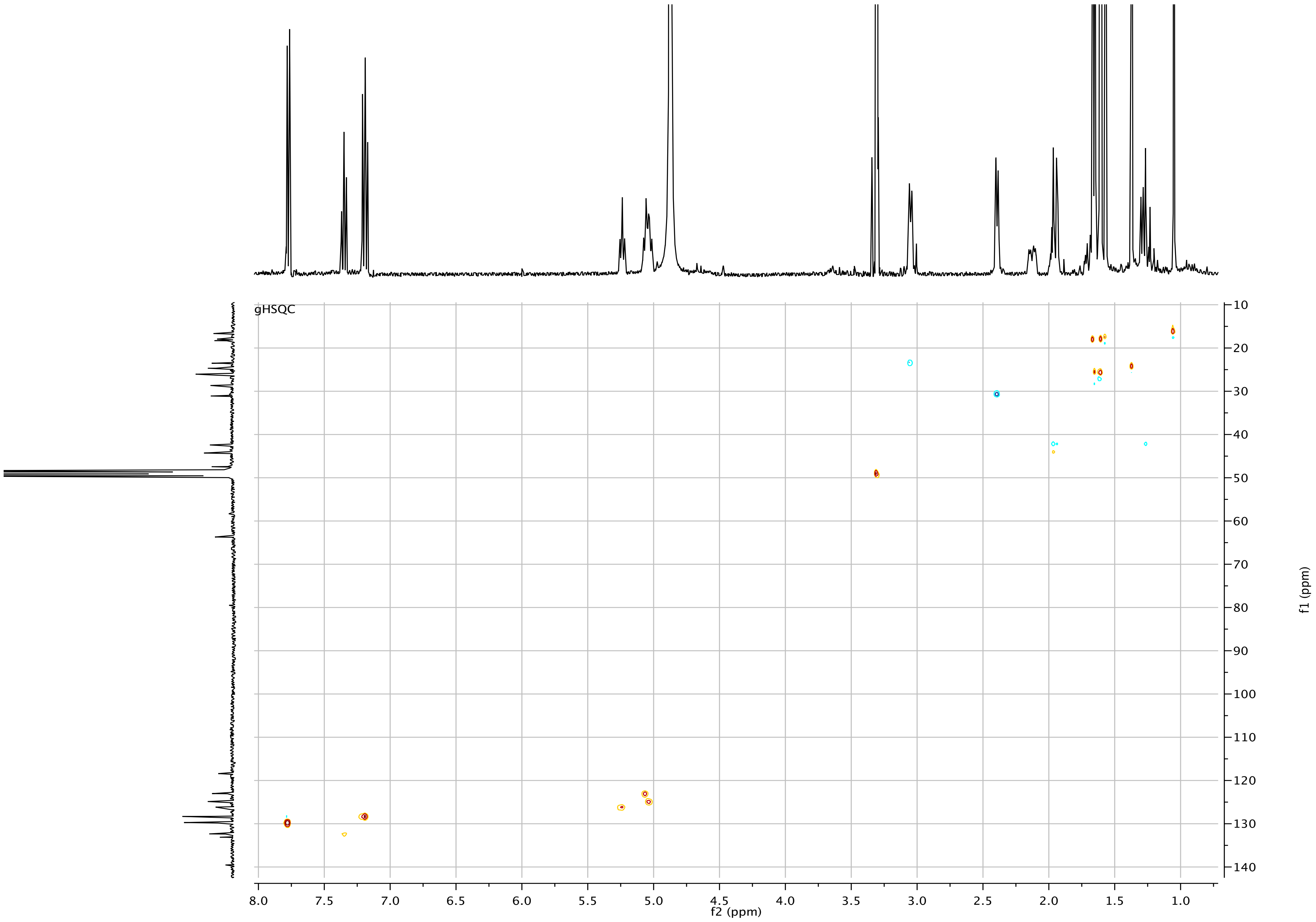

gHSQC
f1 (ppm)
f2 (ppm)

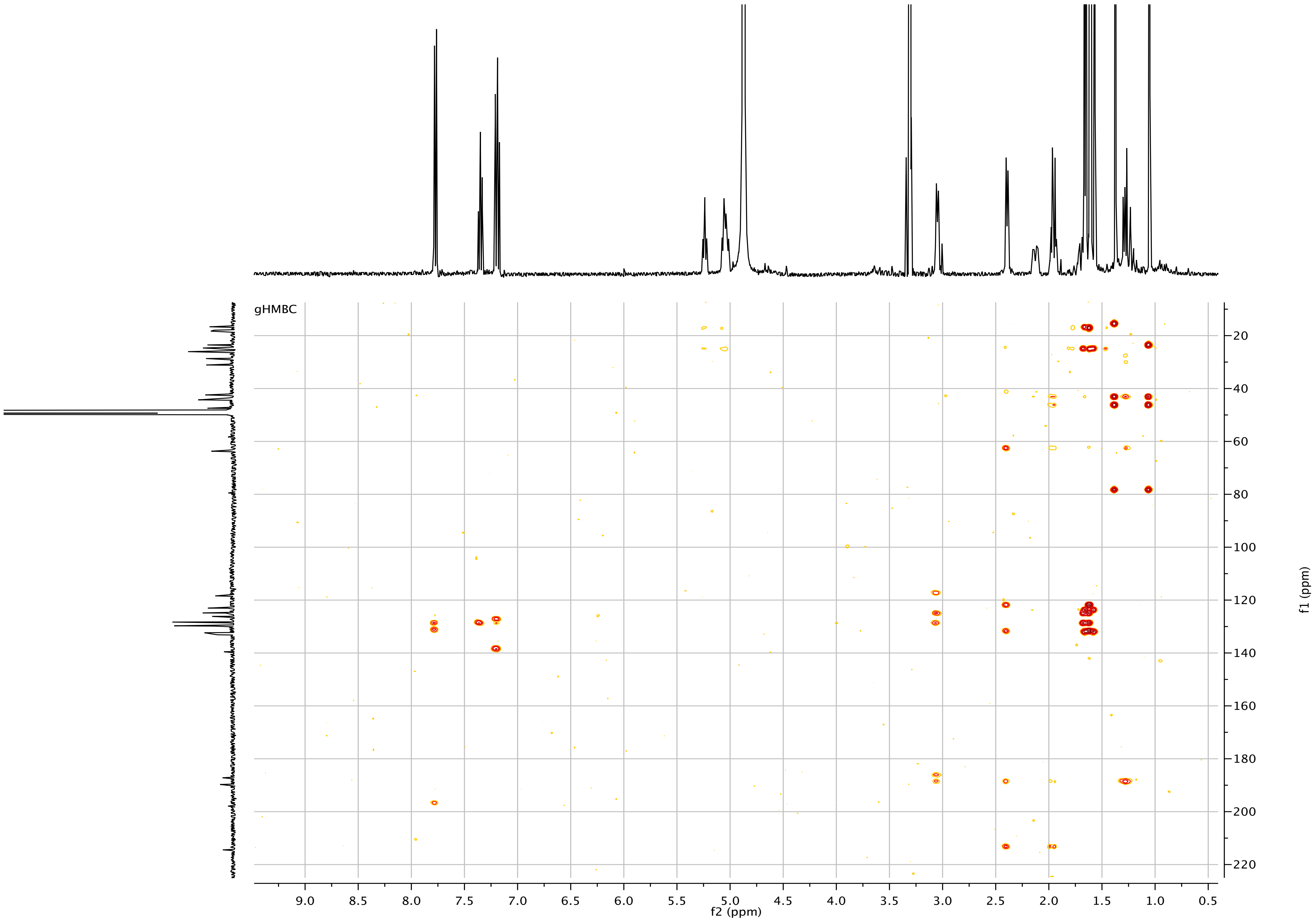

gHMBC
f2 (ppm)
f1 (ppm)

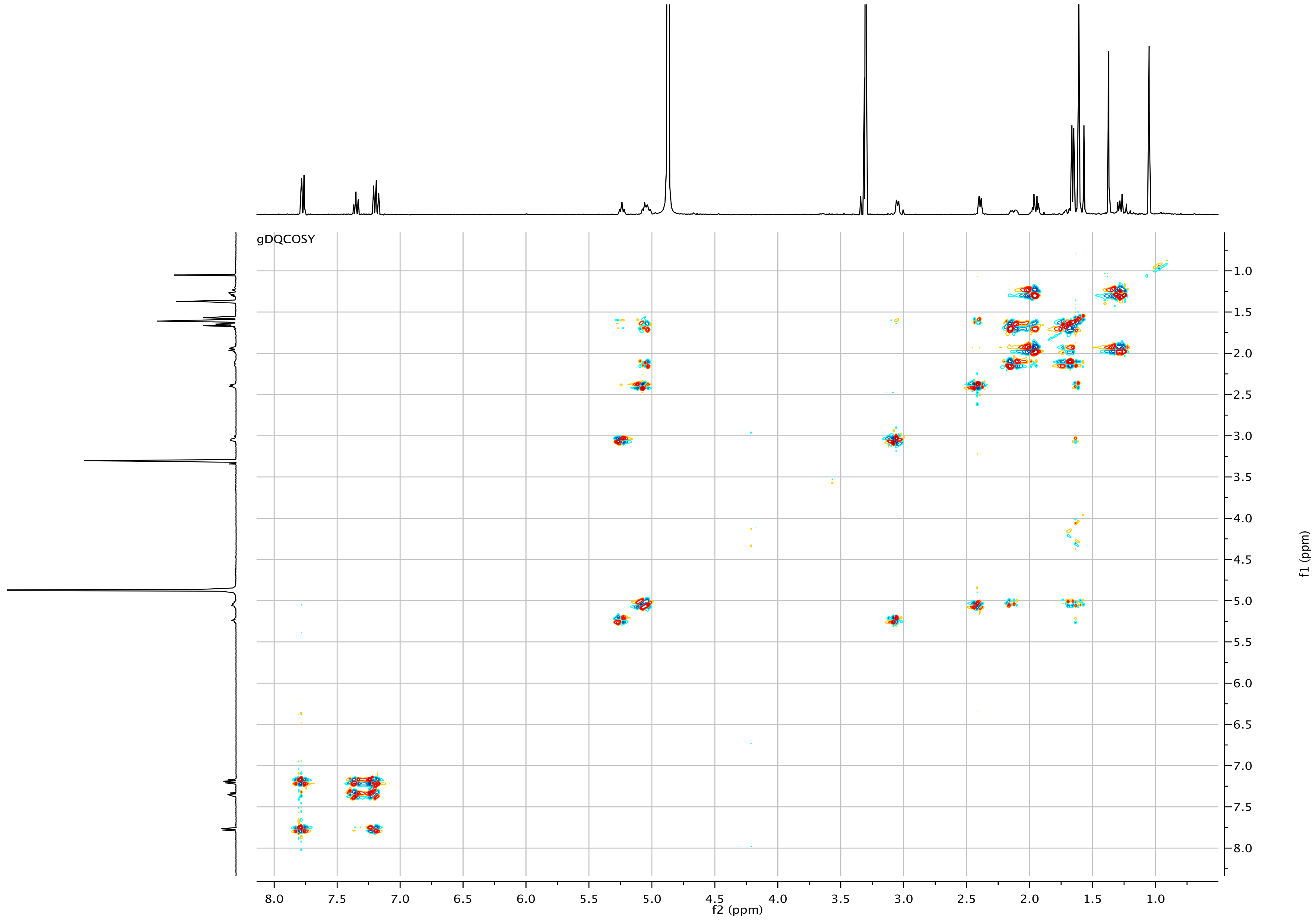

gDQCOSY
f2 (ppm)
f1 (ppm)
8.0
7.5
7.0
6.5
6.0
5.5
5.0
4.5
4.0
3.5
3.0
2.5
2.0
1.5
1.0

Single Mass Analysis

Tolerance = 10.0 PPM / DBE: min = 1.0, max = 50.0
Element prediction: Off
Number of isotope peaks used for i-FIT = 3

Monoisotopic Mass, Even Electron Ions
86 formula(e) evaluated with 1 results within limits (up to 200 best isotopic matches for each mass)
Elements Used:
C: 0-100 H: 0-200 O: 0-15
TAMAYOG/PTOYECTO VIRAL
0808_2012_nemo_V+_2 942 (9.192) 1: TOF MS ES+
3.81e+003

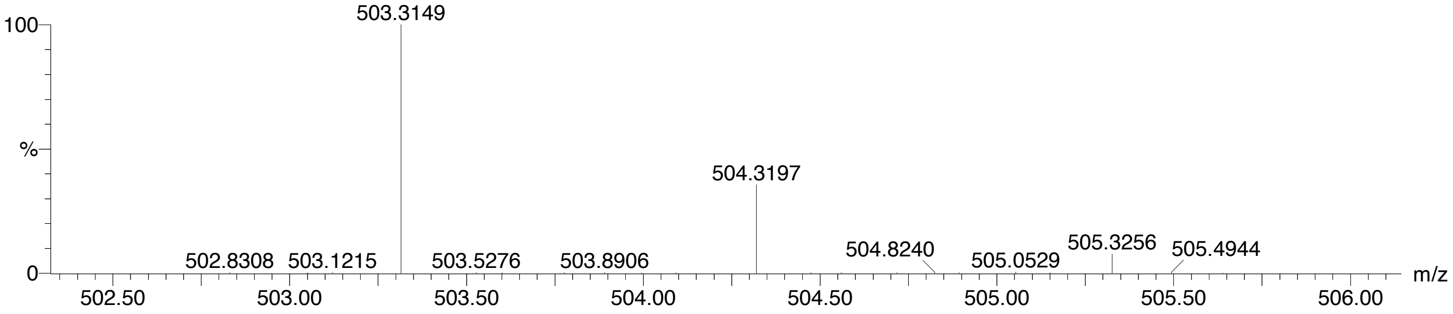

Minimum:				1.0			
Maximum:		5.0	10.0	50.0			
Mass	Calc. Mass	mDa	PPM	DBE	i-FIT	i-FIT (Norm)	Formula
503.3149	503.3161	-1.2	-2.4	12.5	59.7	0.0	C33 H43 O4

Single Mass Analysis
Tolerance = 10.0 PPM / DBE: min = 1.0, max = 50.0
Element prediction: Off
Number of isotope peaks used for i-FIT = 3

Monoisotopic Mass, Even Electron Ions
82 formula(e) evaluated with 1 results within limits (up to 200 best isotopic matches for each mass)
Elements Used:
C: 0-100 H: 0-200 O: 0-15
TAMAYOG/PTOYECTO VIRAL
0808_2012_nemo 1851 (9.338) 1: TOF MS ES- 2.80e+003

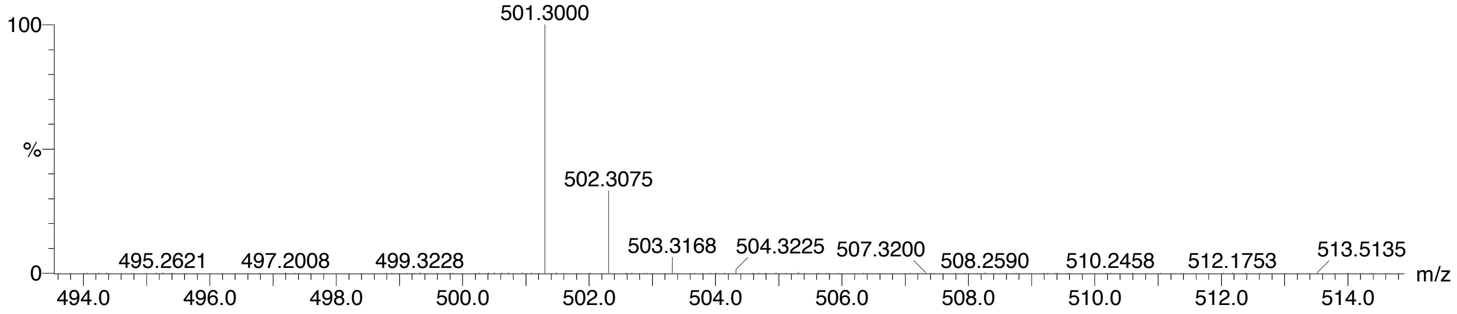

Minimum:				1.0			
Maximum:		5.0	10.0	50.0			
Mass	Calc. Mass	mDa	PPM	DBE	i-FIT	i-FIT (Norm)	Formula
501.3000	501.3005	-0.5	-1.0	13.5	28.1	0.0	C33 H41 O4
